# Contribution of Lower Extremity Joints on Energy Absorption during Soft Landing

**DOI:** 10.3390/ijerph18105130

**Published:** 2021-05-12

**Authors:** Akihiro Tamura, Kiyokazu Akasaka, Takahiro Otsudo

**Affiliations:** 1 Department of Physical Therapy, School of Health Sciences at Narita, International University of Health and Welfare, Narita, Chiba 286-8686, Japan; a.tamura.dp@gmail.com; 2 Department of Physical Therapy, Saitama Medical University Graduate School of Medicine, Moroyama, Saitama 350-0496, Japan; otsudo@saitama-med.ac.jp; 3 School of Physical Therapy, Saitama Medical University, Moroyama, Saitama 350-0496, Japan

**Keywords:** injury prevention, angular impulse, biomechanics, shock attenuation

## Abstract

Soft landing after jumping is associated with the prevention of lower extremity injuries during sports activities in terms of the energy absorption mechanisms. In this study, the contribution of lower extremity joints during soft landing was investigated. Subjects comprised 20 healthy females. Kinetics and kinematics data were obtained during drop vertical jumps using a three-dimensional motion analysis system. Negative mechanical work values in the lower extremity joints were calculated during landing. A multiple regression analysis was performed to determine which lower extremity joints contributed more in achieving soft landing. The means of mechanical work of the hip, knee, and ankle in the sagittal plane were −0.30 ± 0.17, −0.62 ± 0.31, and −1.03 ± 0.22 J/kg, respectively. Results showed that negative mechanical work in the hip and knee is effective in achieving soft landing. These findings indicate that energy absorption in the hip and knee joints might be an important factor in achieving soft landing, whereas that in the ankle has a negative effect. Therefore, when improving soft landing techniques, we should consider energy absorption in the hip and knee via eccentric activation of the hip and knee extensors during landing.

## 1. Introduction

Soft landing after jumping is associated with the prevention of lower extremity injuries during sports activities [1,2]. It is achieved by increasing the hip and knee flexion angle during landings [1,3,4]. Previous studies have reported that landing with a decreased knee flexion angle may increase anterior cruciate ligament loading by increasing quadricep muscle activations and knee extensor moment [2,5]. This finding has been supported by some cadaver studies, where it was revealed that quadricep loading with the slight knee flexion angle causes significant anterior tibial translation and higher tension in the anterior cruciate ligament [6,7,8,9]. Furthermore, knee compression force during soft landings is smaller than that during stiff landings [10,11], possibly decreasing the mechanical stress on bone and cartilage in the knee joint. Therefore, landing with increased knee flexion angles associated with soft landings may be effective in reducing the potential for injuries of the knee joint, including those to the anterior cruciate ligament.

Several studies have examined the energy absorption strategy for the lower extremities during some landing maneuvers [10,11,12,13]. During a normal landing maneuver, the hip, knee, and ankle joints are the impact absorber, and the impact of landing could be absorbed actively by eccentric muscle contraction in the lower extremity joints [11,14]. In particular, the knee joint is the largest contributor of the lower extremity on the energy absorption during landing [13,14]. DeVita and Skelly have reported that soft landing, defined as a knee flexion angle greater than 90 degrees after landing from a vertical height of 59 cm, resulted in a lower vertical ground reaction force (vGRF), as well as impulse and hip extensor and knee extensor moments compared with stiff landings [11]. Results have indicated that landing with increased knee flexion could absorb more impact during landing with the hip and knee joints [11]. These findings suggest that the improvement of landing maneuvers may increase the capacity of the energy absorption in the lower extremities. In addition, evaluating energy absorption in the lower extremity may provide useful information for the improvement of landing techniques.

Prevention programs for anterior cruciate ligament injury are widely conducted for injury prevention or rehabilitation before returning to sport [15,16,17,18]. In recent years, some prevention programs for anterior cruciate ligament injuries have included exercises with a soft landing [19,20]. Furthermore, a clinical screening tool for identifying potentially high-risk movement patterns related to anterior cruciate ligament injury can identify the amount of knee flexion displacement, which achieves soft landing as the main outcome measure [21]. These attempts indicate that the usefulness of soft landing in preventing knee injuries has been widely recognized. Although studies on the efficacy of soft landing in preventing knee injuries have been conducted, most previous reports have only measured kinetics and kinematics in the lower extremity joints during a soft landing [2,5,10]. To learn a soft landing technique, it is necessary to reveal the contribution degree in the lower extremity joints for achieving soft landing compared with a stiff landing. However, it is not clear how respective joints contribute in achieving soft landing compared with a stiff landing. This study aimed to investigate the contribution of lower extremity joints on soft landing with deep knee flexion. This finding could provide effective importations when considering safe landing alignment in injury prevention or rehabilitation before returning to sport. It was hypothesized that the energy absorption in the knee or hip joints during landing is the largest contributor for landing with deep knee flexion, particularly when producing a soft landing.

## 2. Materials and Methods

### 2.1. Participants

Twenty healthy female college students recruited from October 2016 to January 2017 were included in this study (Table 1). A priori power analysis using G*Power version 3.1 indicated that a minimum of 6 participants in each group and a total of 12 participants were needed to obtain α = 0.05, and 1−β = 0.80 based on the knee kinetics variable in our pre-research. Participants were recruited by sending e-mails with research explanatory documents to all students in the university. The students who confirmed their intention to participate were required to answer a questionnaire related to their physical characteristics, current medical information, and medical history. The inclusion criteria included age > 18 years and absence of current pain in the lower extremities during their daily activities. The exclusion criteria included any history of orthopedic trunk, hip, knee, or ankle surgeries and serious injuries. The study was carried out in accordance with the Declaration of Helsinki and was approved by the ethics committee of the university before this study. All participants gave written informed consent for participation prior to testing.

### 2.2. Experimental Design

All experiments in this study were conducted in the motion analysis laboratory of the university. All participants were asked to wear closely fitting dark shorts for data collection. Two force plates (MSA-6 Mini Amp, AMTI, Watertown, MA, USA) were used to record the vertical ground reaction force (vGRF) at a sampling rate of 1200 Hz. A three-dimensional motion analysis system with eight cameras (Vicon MX system, Oxford Metrics, Oxford, UK) was used to record lower extremity kinematics data during testing. Thirty-three reflective markers were placed on the following anatomical and technical landmarks: at the front of the head (bilateral), back of the head (bilateral), seventh cervical vertebra, tenth thoracic vertebra, clavicle, sternum, right back, shoulders, lateral epicondyles of the elbows, medial wrists, lateral wrists, second metacarpal heads, anterior superior iliac spines, posterior superior iliac spines, lateral thighs, lateral epicondyles of the knees, lateral tibias, lateral malleoli, second metatarsal heads, and heels. All marker positions were sampled at 240 Hz and low-pass filtered at 16 Hz with a fourth-order zero lag Butterworth filter. A Vicon Plug-in-Gait Full Body marker set was used to obtain lower extremity kinematics data [22,23]. The Vicon Nexus software (version 1.8.5; Oxford Metrics, Oxford, UK) were used to calculate joint moments and power in the lower extremity based on kinematics and grand reaction force data. Moreover, Microsoft Excel 2013 (Microsoft Corporation, Redmond, WA, USA) was used to manage the data obtained from the Vicon Nexus software.

### 2.3. Data Processing

The participants performed drop vertical jumps (DVJs) from a box that was 40 cm in height [23,24]. The DVJ procedure was done in two stages: the first involved landing after dropping down from the box, and the second involved landing after a maximal vertical jump, landing from the first drop. This technique was set based on our previous study [23] and Hewett et al.’s (2005) [24]. Participants were instructed to jump as high as possible after dropping down from the box and required to land on one force plate with the dominant foot and another force plate with the nondominant foot. Before testing, an assistant teacher explained the sequence with the method of DVJs to all participants, and they were asked to perform several practice trials to become familiar with the method of DVJs. They were asked to repeat subsequent trials until three successful trials were achieved. Participants were excluded from the trial if they lost balance during the landing stages.

During the deceleration phase of the first landing after dropping down from the box, kinetics and kinematics data of the participant’s dominant legs were obtained. The dominant leg of each participant was determined based on which foot was used to kick a ball as far as possible [23,24]. The deceleration phase was defined as the duration from the initial ground contact, which was defined as the moment when the vGRF first exceeded 10 N [23,25,26], to the moment when the maximum angle of knee flexion was recorded during landing [23,27].

During the deceleration phase of landing, each participant’s hip, knee, and ankle angles in the sagittal plane were calculated based on the filtered three-dimensional coordinate data by calculating the Euler angle. The hip flexion, knee flexion, and ankle dorsi-flexion angles represented positive values, whereas the hip extension, knee extension, ankle plantar flexion angles represented negative values. These joint angles were recorded at the point of maximum knee flexion angle, which represents the end point of the deceleration phase of landing. Previous studies have defined two landing techniques (i.e., soft and stiff landing) according to the degree of knee flexion angle during landing [11,28]. This study also divided the participants into two groups (soft and stiff landing groups) according to the mean value of participant’s maximum knee flexion angles during landing. That is, a participant was assigned to the soft landing group if her angles were more than the mean maximum knee flexion angles, and the rest of the participants were classified under the stiff landing group.

The vGRF and joint internal moments of the hip extensors, knee extensors, and ankle plantar flexors were recorded at the two time points of the maximum value of each variable and maximum knee flexion angle, which represent the end point of the deceleration phase of landing. In terms of joint moment, hip extensors, knee extensors, and ankle plantar flexors were assigned positive values, whereas hip flexors, knee flexors, and ankle dorsiflexors were assigned negative values. The joint powers of the hip extensors, knee extensors, and ankle plantar flexors were calculated as the product of instantaneous joint moment and joint angular velocity in each joint. Regarding joint moments and powers, vGRF variables were normalized according to each participant’s body weight (kg).

The mechanical work performed by the net flexor/extensor moments at the hip, knee, and ankles joints was calculated by integrating the joint power curves over time during the deceleration phase of landing. The negative values of the mechanical work represent energy absorption in each joint during the deceleration phase of landing [11,29,30].

### 2.4. Statistical Analysis

The means and standard deviations of the peak joint angles, moments, powers, vGRF, and joint angles at the point of maximum knee flexion were calculated for data analysis, and then unpaired t-tests were carried out to examine the differences of each variable between the soft and stiff landing groups. In addition, negative work values of the lower extremity joints during deceleration were calculated; then, a multiple regression analysis was performed to identify contributors on achieving soft landing, which is defined using the knee flexion angle. The dependent variable was set at the maximum knee flexion angle during landing and the independent variables were negative mechanical works at the hip, knee, and ankle during the deceleration phase of landing. *p* values < 0.05 were considered statistically significant. Data were analyzed using the using the Statistical Package for the Social Sciences software version 24.0 (IBM Corp., Armonk, NY, USA).

## 3. Results

All participants were divided into the soft landing group (*n* = 10) and stiff landing group (*n* = 10) according to their maximum knee flexion angles during landing. The physical characteristics of the participants were as follows: soft landing group: age, 20.8 ± 0.7 years; height, 161.1 ± 3.9 cm; and weight, 55.6 ± 6.0 kg and stiff landing group: age, 21.5 ± 0.6 years; height, 160.0 ± 4.5 cm; and weight, 52.6 ± 7.3 kg (Table 1). No significant differences were observed in terms of the physical characteristics of the groups (*p* > 0.05). The mean maximum knee flexion angle during landing of all participants was 72.67 ± 10.91°. The mean maximum knee flexion angles of the soft and stiff landing groups were 80.99 ± 8.07° and 63.34 ± 5.87°, respectively.

**Figure 1:**
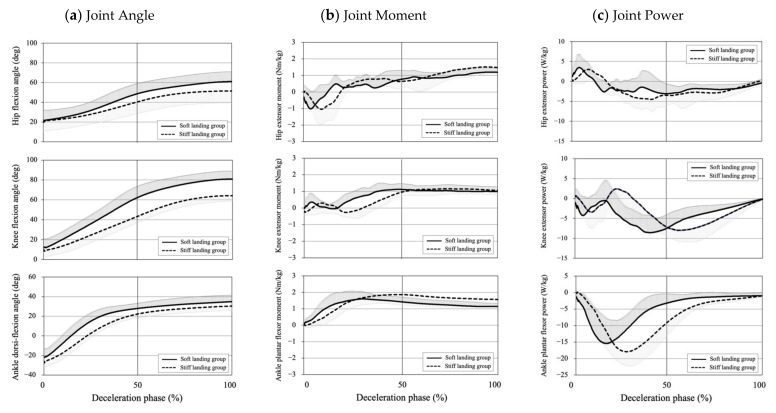
Joint angles, joint moments, and joint powers in the lower extremity joints in the soft and stiff landing groups. These figures represent the means and standard deviations of the hip flexion, knee flexion, and ankle dorsiflexion angles, joint moments (**b**), and powers (**c**) of the soft landing (solid lines) and stiff landing groups (broken lines) during the deceleration phase normalized to 100%. Joint moments and powers were normalized according to the participant’s body weight (kg).

The hip and ankle joint angles at the point of maximum knee flexion angle during landings at two groups were 61.11 ± 9.99° and 51.49 ± 12.26° for the hip flexion angle and 34.89 ± 6.59° and 30.41 ± 4.44° for the ankle dorsi-flexion angle. No significant differences were observed between the two groups in terms of these variables (*p* > 0.05). The patterns of hip, knee, and ankle joint angles during the deceleration phase in the soft and stiff landing groups are shown in Figure 1.

The maximum value of joint angles, moments, powers, and vGRF during landing are shown in Table 2. The ankle dorsiflexion angle in the soft landing group was significantly larger than that in the soft landing group (*p* < 0.05). No significant differences were observed in terms of hip flexion, adduction, and knee abduction angles between two groups. In addition, joint moments: powers in the hip, knee, and ankle joints: and vGRF did not significantly differ between the two groups. The patterns of the hip, knee, and ankle joint moments and powers during the deceleration phase in the soft and stiff landing groups are shown in Figure 1.

The mean mechanical work values of the hip, knee, and ankle in the sagittal plane were −0.30 ± 0.17, −0.62 ± 0.31, and −1.03 ± 0.22 J/kg, respectively. The values of the mechanical work of the hip, knee, and ankle were −0.32 ± 0.19, −0.78 ± 0.27, and −1.02 ± 0.24 J/kg, respectively, in the soft landing group and −0.28 ± 0.15, −0.47 ± 0.25, and −1.05 ± 0.19 J/kg in the stiff landing group, respectively. Based on the results of the multiple regression analysis, mechanical work in the hip, knee, and ankle in the sagittal plane was a significant contributor in achieving a soft landing with flexed knee flexion. The multiple regression model for the prediction of soft landing technique is presented in Table 3. The adjusted multiple R2 was 0.70 and this value was statistically significant (*p* < 0.05). In terms of independent variables, mechanical work values in the hip, knee, ankle joints were statistically significant in all groups (*p* < 0.01). In terms of hip and knee joints, the standardized coefficients had a negative value; that is, increasing negative mechanical work in the hip and knee joints was effective in achieving soft landing with knee flexion, whereas that in the ankle joint was counterproductive. Furthermore, work in the knee joint had a greater coefficient than that in the hip joint when comparing each standardized coefficient.

## 4. Discussion

In this study, we aimed to investigate the contribution of lower extremity joints to soft landing caused by knee flexion in terms of energy absorption mechanisms. To evaluate the amount of energy absorption in the lower extremity joints, mechanical work has been widely used in several related studies [11,14,31,32,33]. In general, the negative values of mechanical work indicate the amount of energy absorption thorough eccentric muscle contraction in the opposite direction to the angular velocity of the joints [29]. When considering lower extremity joints during landing, an increase in the negative mechanical work indicates absorption of the impact at the moment of landing by decelerating the flexion motion of the lower extremity joints via eccentric muscle contraction (i.e., hip extensor, knee extensor, and ankle plantar flexor muscles). Therefore, the change in negative mechanical work can interpret energy absorption strategy during the deceleration phase of landing. In contrast, the positive values of mechanical work, namely, the positive mechanical value, indicate the amount of power generation thorough concentric muscle contraction for the same direction as the angular velocity of the joints [29]. Generally, this is observed in the lower extremity joints at the propulsion phase of jumping [34].

The results of this study showed that negative mechanical work of the hip and knee joints is a greater contributor for producing soft landing during the deceleration phase. Thus, energy absorption in the hip and knee joints may be important factors in achieving soft landing. Previous studies have indicated that the knee joint was the most important impact absorber in the lower extremities during normal landing [14,31,35]. Results of the present study support such a conclusion. In addition, the hip joint is a key factor for energy absorption during landing in females who land with knee valgus alignment, which could be a risk factor of the ACL injury [21]. In general, hip extensors, e.g., gluteus maximus, are more likely to generate during the deceleration phase after landing from jumps [36,37]. Zazulak et al. have reported that decreased hip extensors and increased knee extensor activities may be important contributors for the increased susceptibility of non-contact ACL injuries in female athletes [38]. Therefore, energy absorption in the hip joint may be important in preventing knee injuries. When considering improving the soft landing technique, we should consider not only energy absorption in the knee joint, but also that in the hip joint via eccentric activation of the hip extensor during the deceleration phase of landing. With regard to the prevention of non-contact ACL injuries, these injuries commonly occur with sudden mechanical stress applied to the knee joint during landings [39]. In addition, landings with a slightly flexed knee joint may cause anterior tibial translation and higher tension of the anterior cruciate ligament [6,7,8,9]. Therefore, the improvement in the soft landing by promoting the energy absorption in the hip and knee joints could reduce the risk of knee injury or maintain orthopedic conditions of the knee joint.

Regarding energy absorption in the ankle joint, the present study showed that the ankle joint was a negative contributor for energy absorption in achieving a soft landing. Previous studies have reported that that ankle joint was also one of the contributors for energy absorption during the deceleration phase [11,14,40]. DeVita et al. have reported that the ankle joint was the largest contributor for energy absorption in the lower extremity joints during stiff landing [11]. Furthermore, they have concluded that the relative contribution of the ankle joint increased, whereas those of the hip and knee joints decreased [11]. In the present study, a negative mechanical work in the ankle joint was greater than that in the hip and knee joints, as with the stiff landing condition by DeVita et al. [11]. In addition, achievement of soft landing was affected by the decrease of contribution in the ankle joint. This finding indicated that the stiff landing may reduce the capacity of energy absorption in the hip and knee joints by depending on that in the ankle joint. The energy absorption in lower extremity joints was controlled by adjacent joints [11]. In particular, the energy absorption in the hip joint could be assisted by the movement at the trunk and knee, and that in the knee joint could be assisted by the movement at the hip joint and ankle. In contrast, the ankle is potentially required to have a large energy absorption capacity because it is assisted only by the movement at the knee joint. Therefore, although the energy absorption in the ankle joint was greater than that in the hip and knee joints, the energy absorption in the ankle joint may be distributed to the knee joint by achieving soft landing.

The difference between the soft and stiff landing groups, which were defined according to the mean maximum knee flexion angles during landing, was significant only in the peak ankle dorsi-flexion angle. A greater ankle dorsiflexion during soft landing was caused by anterior tilting of the tibia with flexed knee joint while feet were fixed to the ground surface during landing. Some reports have also shown that an increased knee flexion angle is associated with an increased ankle dorsiflexion angle [41,42]. Therefore, results of this study were in accordance with those of previous reports [41,42]. Regarding other joint angles, peak hip flexion, adduction, and knee abduction angles did not significantly differ between the soft and stiff landing groups. In addition, joint moments, powers, and vGRF did not differ. Thus, based on the results of this study, achieving the soft landing did not affect hip and knee frontal joint kinematics and lower extremity joint kinetics. Furthermore, vGRF also did not significantly differ between the soft and stiff landing groups. This result indicated that the landing movement was performed by compensating for energy absorption capacity in the respective joints. Therefore, achieving soft landing could alter the energy absorption strategy in the lower extremity without altering total impact force from the ground.

For injury prevention or rehabilitation before returning to sport, the findings of this study suggest the need to improve the landing alignment for soft landing and achieve soft landing. Although most previous ACL injury programs [19,20] and a clinical screening tools [21] have focused on soft landing during exercises, in general, instructors have provided verbal instructions that the knee or hip joints of the athletes should be bent more during landing. Our study provides evidence that the energy absorption capacity of the hip and ankle joints is an important factor in achieving soft landing. In the future studies, some exercises promoting eccentric hip and knee extensor activations should be considered for the improvement of an individual’s landing technique related to energy absorption capacity during landing.

This study had limitations. Healthy female participants who had a history of the ACL injury or other knee injuries were not included in this study. Thus, the sample size was small. Females who had a history of the ACL injury have different landing alignments and muscle activations in the lower extremity compared with healthy females [43,44,45]. Therefore, in future studies, we should evaluate the characteristics of landing alignment and muscle activations related to the energy absorption strategy in females after ACL injuries for the prevention of the ACL injury and re-injury.

## 5. Conclusions

Results of the present study showed the contribution of lower extremity joints on soft landing caused by flexed knee joints in terms of energy absorption mechanisms. Energy absorption in the hip and knee joints was a greater contributor for achieving soft landing, which was defined according to knee flexion angle. In addition, energy absorption in the ankle joint negatively affected the achievement of soft landing. These findings indicate that energy absorption in the hip and knee joints were important factors in achieving soft landing and that in the ankle it was counterproductive. Improving the soft landing technique by promoting energy absorption in the hip and knee joints may reduce the risk of knee injury or maintain orthopedic conditions of the knee joint.

## Figures and Tables

**Table 1:** Participants’ information ^a^.

	All Participants	Soft Landing Group	Stiff Landing Group	*p* Value ^b^
Individuals	20	10	10	-
Age (years old)	21.0 (0.9)	20.8 (0.7)	21.5 (0.6)	>0.05
Height (cm)	160.4 (3.9)	161.1 (3.9)	160.0 (4.5)	>0.05
Weight (kg)	53.6 (6.3)	55.6 (6.0)	52.6 (7.3)	>0.05

^a^ Data are presented as mean (± standard deviation). ^b^ *p* value comparing the soft and stiff landing groups.

**Table 2:** The means maximum value of lower extremity joint angles, moments, powers, and vGRF during the deceleration phase of landings in the soft and stiff landing groups ^a^.

Title	Soft Landing Group (*n* = 10)	Stiff Landing Group (*n* = 10)	*p* Value ^b^	Effect Size ^e^
Joint Angles (degree)				
Hip Flexion	61.61 (9.87)	51.87 (12.25)	0.08	0.87
Hip Adduction	3.73 (6.19)	0.01 (5.47)	0.19	0.63
Knee Flexion	80.99 (8.07)	63.34 (5.87)	0.00 ^c^	2.39
Knee Abduction	−2.60 (2.94)	−3.18 (3.90)	0.72	0.10
Ankle Dorsi-Flexion	37.10 (5.30)	31.56 (5.10)	0.04 ^d^	1.07 ^c^
Joint Moment (N∙m/kg)				
Hip Extensor	1.90 (0.52)	2.23(0.57)	0.22	−0.59
Hip Abductor	0.79 (0.45)	0.79 (0.31)	0.98	0.01
Knee Extensor	1.45 (0.32)	1.39 (0.40)	0.72	0.17
Knee Adductor	0.40 (0.21)	0.47 (0.25)	0.56	−0.28
Ankle Plantar Flexor	1.88 (0.31)	2.16 (0.49)	0.16	−0.69
Joint Power (W/kg)				
Hip Extensor	−9.18 (4.63)	−10.61(4.56)	0.52	0.31
Hip Abductor	−2.40 (1.20)	−3.44 (2.81)	0.32	0.48
Knee Extensor	−13.18 (3.82)	−11.49 (3.26)	0.33	−0.48
Knee Adductor	−1.79 (0.87)	−1.72 (0.99)	0.88	−0.07
Ankle Plantar Flexor	−18.23 (5.61)	−19.40 (4.31)	0.62	0.23
vGRF (N/kg)	2.00 (0.53)	2.37 (0.38)	0.11	−0.79

Abbreviations: vGRF, vertical ground reaction force. ^a^ Data are presented as mean (± standard deviation). ^b^ *p* value comparing the soft and stiff landing groups. ^c^ Statistically significant at *p* < 0.001. ^d^ Statistically significant at *p* < 0.05. ^e^ Effect sizes were calculated using the Cohen d.

**Table 3:** Multiple regression analysis of joint works in the lower extremity during the deceleration and take-off phase of landing (R^2^ = 0.74, adjusted R^2^ = 0.70).

	Unstandardized Coefficients		Standardized Coefficients	t Value	*p* Value	95% CI ^a^
	B	B SE	β			
Hip Joint Work	−28.85	8.29	−0.57	−3.47	0.003 ^b^	−46.43, −11.27
Knee Joint Work	−32.8	5.11	−0.64	−6.42	0.000 ^b^	−43.66, −21.97
Ankle Joint Work	31.01	7.20	0.61	−4.31	0.001 ^b^	15.75, 46.28

^a^ CI: confidence interval. ^b^ *p* values < 0.01 were considered statistically significant.

## Data Availability

The data sets used and analyzed during the current study are available from the first author.
